# Geographical data for the definition of theoretical STEMI patient base for hospital emergency departments in the Lazio region, Italy

**DOI:** 10.1186/1472-6963-14-S2-P73

**Published:** 2014-07-07

**Authors:** Francesca Mataloni, Mariangela D’Ovidio, Mirko Di Martino, Paolo Sciattella, Marina Davoli, Danilo Fusco

**Affiliations:** 1 Department of Epidemiology, Lazio Region Health Service, Rome, Lazio, Italy

## Background

Spatial analysis and geographical data have been used increasingly in recent years to identify the relationship between supply and demand in Health Services. The aim of the study is to define a theoretical STEMI patient base for hospital Emergency Departments (ED) of the Lazio region.

## Methods

Theoretical STEMI patient bases were defined taking into account 4 parameters:

• travel time (minutes), from each census-block of the region to each ED, during the night and during the most traffic congested hours;

• the median waiting time between the access and taking care of patients with white, green and yellow triage were calculated for each ED, as a proxy of the ED’s workload;

• the number of hospital admissions in 2012, as a proxy of hospital size

• % of patients treated with PCI within 90 minutes, as a proxy of hospital performance.

All parameters were used to assign a score to each ED and a weight was assigned to each parameter. A weighted average was calculated and each census-block was assigned to the ED with the highest score. Moreover, two constraints were established: time travel from census-block to ED must be lower than 20 minutes; ED must have hemodynamic equipment.

## Results

In the Lazio region there are 31,988 census-blocks and 50 hospital ED (46 of them are generic and 20 with hemodynamic). 27% of the regional population (10,473 census-blocks) do not reach an ED hospital with hemodynamic in 20 minutes during the night; these census-blocks are mainly situated outside the city of Rome. Taking into account the most traffic congested hours during the day, the percentage of regional population not covered by an adequate health service increased to 42% (15,062 census-blocks), including some census-blocks located in the city of Rome.

## Conclusions

Geographic data and travel time from home to hospital are useful to identify theoretical STEMI patient bases, data on traffic congested hours should be considered for a better identification of the population not covered by an adequate health service.

